# 

**Published:** 1897-07

**Authors:** 


### INDEX TO VOL. XIII.

American Academy of Railway Surgeons.......................... 196
Another Medical College in Texas .................................. 85
Acute Glanders....................................................... 124
Apologetic............................................................ 94
Abortion, A Case of, with Treatment.............................. 7
Afraid of Microbes.................................................. 245
Alcohol or Opium..................................................... 284
Abstracts and Selections..........76-83, 399-414, 463-466, 519-524
Antitoxin.............................................................. 295
Anonymous Editorial.... ............................................ 302
Anæmia..... ........................................................... 388
Absence of [illegible] ....................................................... 388
American Me[illegible]cal Association...................................460, 529
Abdominal Section in the Country .................................. 505
Announcement and Program Texas State Medical Association 508
American Medical Association—Committee of Arrangements 515
Advertising Physicians and the Code .............................. 525
Abscess of Both Ovaries—Rupture and Death.................. 560
Annual Meeting of the Texas State Medical Association..... 594
American Medical Association—Denver Meeting ............. 578
A Lively Session...................................................... 632
Anatomical Changes in Malarial and Yellow Fever............ 610
Association of Physicians and Surgeons of Bell County...... 626

Big Tongue..................... ........................................ 27
Blessing in Disguise.................................................. 35
Brazos Valley Medical Society.................................. 196, 222
Book Notices.........51-53, 99-101, 205-207, 257-259, 307-309
370-373, 423-426, 475-478, 533-539, 591-595............641-645
Big Tongue............................................................ 224
Brazos Valley Medical Association ......................... 290, 624
Blanche, Tray and Sweetheart..................................... 301
Bully .............................................. ........... 302
Beaumont Meeting.................................................. 365

Bill for the Doctor's Benefit .......... 395
Burt Case .......... 469
Bovinine Company .......... 528

Central Texas Medical Association .......... 28
Competition for the Senn Medal .......... 30
Congress of the National Prison Association .......... 134
Clinical Experience with Formaldehyde .......... 142
Case at Galveston—A Correction .......... 302
Communication from Committee on Section on Medicine, State Medical Association .......... 389
Complicated Case of Obstetrics .......... 448
Cock's Anti-Bacilli Compound .......... 471
Committee of Arrangements' Announcement .......... 514
Correction of Spinal Deformity by Manual Force .......... 527

Dentition and Diarrhœa .......... 68
Dr. Bibb and the Mexican National Railroad .......... 87
Death of Mrs. Sholars .......... 93
Dallas School of Pharmacy .......... 128
Doctors .......... 138
Difference, The .......... 139
Duties of the Medical Association .......... 144
Demon Alcohol .......... 211
Doctors' Tax .......... 237
Death of Dr. Bratton .......... 249
Dr. Fisher's Reply to an Editorial in the "Texas Medical Journal" .......... 276
Department of Public Health .......... 292
Dr. Fisher .......... 297
Death of Dr. Blackburn .......... 305
Diphtheria and Its Treatment with Antitoxin .......... 445
Dr. Bibb .......... 469
Death of Dr. Quimby .......... 636
Decadence of the State Medical Association—Why? .......... 630

East Texas Medical Society .......... 30
Endarthritis Deformans .......... 55
East Texas Medical Association .......... 283
Epidemic of Experts .......... 361
Eclampsia—A Case of .......... 387

Explanatory and Apologetic.......... 417
Examination of Surgeons .......... 588

For Sale ..........369-370, 426-427, 478-479

Government Investigation of Adulteration.......... 234
Gallinger Bill.......... 470

Hospital Enterprise .......... 143
Has Medicine Advanced?.......... 1
History of the Pathology of Tuberculosis Pulmonary Phthisis from Laennec to Koch.......... 183
Hæmaturia.......... 286
Higher Education ..........76-83
Halt Called.......... 88

International Medical Congress of Moscow.......... 225
Insane Asylums.......... 579
Injuries of Parturition.......... 579
Important Announcement.......... 627
"Insurance," "Assurance" and "Insolence".......... 622

Jurisprudence of Insanity, with Especial Reference to the Case and Trial of Eugene Burt.......... 157

Letter from Dr. Reeves.......... 223
Louisiana State Board of Health.......... 344
Lithotomy .......... 377
Letter from Dr. G. S. West, Palestine, Texas.......... 390
Letter from Dr. Wolff, Brownsville, Texas.......... 396
Late Epidemic.......... 418
Lavaca County Medical Society.......... 463
Late Quarantine Convention.......... 467
Loud Bill, The.......... 470
Leprosy.......... 583

McLaughlin, Dr. J. W., Appointment of.......... 200
Medical News and Miscellany..........41-51, 94-98, 149-150, 203-205, 250-257, 306-307, 638-399, 420-423, 472-474, 530-533, 589-591..........637-640
Malarial Question—Letter from Dr. Denman .......... 25
Medical College Craze.......... 72

Medical Practice in New Mexico.................................... 215
Marlin Wonder.......................................................... 244
Murphy at Moscow....................................................... 245
Malaria.................................................................. 380
Malcastian Mallities Osseum, Osteo Malacia.................. 433
Malarial Hæmaturia..................................................... 501
Mississippi State Medical Association............................ 519
Mortality in Cuba and the Necessity for its Acquisition by the United States........................................................ 585

New Method of Treating General Suppurative Peritonitis.. 146
National Prison Association........................................... 195
Necrological............................................................. 246
Newer Antiseptics....................................................... 297
Neurosis of the Stomach from a Surgical Standpoint........ 319
Nasal Catarrh with Especial Reference to the Correction of the Fetor Ozæna.................................................... 356
Nigger in the Woodpile................................................ 420
New York Academy of Medicine.................................... 455
North Texas Medical Association................................... 626

Ode to Texas Doctors.................................................. 510
Outrage on Texas........................................................ 129
Organizing............................................................... 304
Opportunities and Responsibilities of the Medical Man of Today................................................................... 16
Operation for Strangulated Hernia Upon a Man 80 Years of Age.................................................................. 217
Organization Texas Medical Profession........................... 250
Open Letter.............................................................. 398
Other Objectionable Ways of Advertising........................ 526
Oil of Turpentine in Post-partum Hemorrhage.................. 601

Power of Topical Blood Supply in Gonorrhœa.................. 33
Publishers' Notes.........53-54, 101-104, 150-156, 207-210, 259-264, 312-318, 371-376, 428-432, 481-488, 539-544, 595-600.........................................................645-648
Phonendoscope and Its Use........................................... 59
Poisoning by Filix Mas................................................ 115
Protest Against the Doctors' Tax.................................... 359
Paralysis of the Intestines After Abdominal Operations..... 578

Production of Plasmatic Cell Juices.................................. 580
Personal.................................................................. 627
Pyrexia and Hyperpyrexia.............................................. 621

Quarantine Convention .................................................. 415
Quarantine Convention at Mobile, Ala............................. 449
Quarantined Patients Without Food................................ 580

Reciprocity in Medical Licensure.................................... 31
Rocky Mountain Interstate Medical Association............... 71
Relation of Federal to State Quarantine ......................... 105
Relation of the Nasal Mucosa to the Genitals.................... 119
Radiant Heat in Ulcers of the Leg.................................. 147
Report of a Case of Strangulated Hernia......................... 220
Rabies...................................................................... 230
Report of a Case of Membraneous Croup Successfully Treated with O'Dwyer's Tubes............................... 556

Some Practical Observations in Pelvic Operations............ 9
Some Overlooked Points in Liver Troubles...................... 21
Society Notes............................................................. 70
Specific Action of Quinine in Malaria............................ 148
Sleeplessness............................................................. 190
South Texas Medical Association.............................221, 283
Syphilis.................................................................... 489
Suprapubic Cystotomy—Some Complications.................. 116
Surgical Cases Occurring in the Practice of a Country Doctor ..................................................................... 499
Some Perils of Child-bearing, and Their Prevention.......... 545
Static Electricity as a Curative Agent—Report of Cases..... 604

Therapeutic Notes..................98-99, 309-312, 427-428, 479-480
To Practice Medicine in Texas........................................ 37
To Our Subscribers..................................................... 201
To Investigate Yellow Fever .......................................... 237
Timely Words............................................................ 91
Trip to Mexico........................................................... 91
Trials of a Young Doctor .............................................. 112
Those Vacant Chairs................................................... 136
Tri-State Medical Society ............................................. 296
Treatment of Eczema With Picric Acid............................ 233
Taxing the Doctors: A Protest....................................... 243

Treatment of Malignant Mammary Neoplasma .................. 265
Tuberculosis of the Bones and Joints............................. 272
Texas to be Congratulated............................................ 294
Texas Physicians.......................................................... 364
Thing of Beauty ........................................................... 366
Texas State Medical Association ...........................462, 468
Texas State Medical Association—President's Announcement............................................................ 517
Texas State Medical Association—Section on Surgery........ 518
Typho-Malaria.............................................................. 468
Typhoid Fever.............................................................. 562
The Texas Eye, Ear, Nose and Throat Charity Hospital..... 588
The Burt Case.............................................................. 633
The Transactions.......................................................... 629
The Need of and How to Secure Medical Legislation in Texas.......................................................................... 616

Unilateral Salpingo Oophorectomy and Appendicectomy, with Removal of Four Calculi from the Transverse Mese-colon ........................................................... 608

Volume Thirteen—Our Thirteenth Voyage....................... 40

Who is Responsible?..................................................... 197

Yellow Fever Bacillus ................................................... 192
Yellow Fever Experts and Things.................................. 239
Yellow Fever................................................................ 471

